# Use of Anterior Plates in Anterior Cervical Discectomy and Fusion After Traumatic Fracture Dislocations Lead to Early Adjacent-Level Degeneration

**DOI:** 10.7759/cureus.94629

**Published:** 2025-10-15

**Authors:** Mariana Otero, Joyita Barua, Purnajyoti Banerjee

**Affiliations:** 1 School of Medicine and Dentistry, Queen Mary University of London, London, GBR; 2 Department of Neurosurgery, The Royal London Hospital, London, GBR; 3 Department of Orthopaedics, The Royal London Hospital, London, GBR

**Keywords:** anterior cervical discectomy and fusion (acdf), complications, disc degeneration, spine, trauma

## Abstract

Objectives

Adjacent-level disc degeneration after anterior cervical discectomy and fusion (ACDF) is established in degenerative cervical disease. Plate-to-disc distance (PDD) <5 mm is a known cause of this complication. Our aim was to assess the likelihood of disc degeneration of adjacent levels in patients with traumatic fracture dislocation within two years of surgery.

Methods

We retrospectively reviewed scans of patients who underwent ACDF surgery using an anterior plate for fracture dislocation of the cervical spine in the Royal London Hospital, London, UK. ACDF for indications other than trauma and fusions without an anterior plate were excluded. The distance between the edges of the plate and the superior and inferior intervertebral discs was measured on scans before and after surgery, with six months to two years of follow-up. Degeneration of the adjacent intervertebral discs was assumed if there was a difference in height equal to or greater than 30% and signs of ossification.

Results

Twenty-five patients with 50 discs were included. We investigated a PDD cutoff of 5 mm. Adjacent discs with PDD >5 mm were in group A (n = 14) while those with PDD <5 mm were in group B (n = 36). The mean age was 38 and 43 years, respectively. There was significantly lower incidence of radiological evidence of disc degeneration in group A compared to group B (43.24% vs. 15.38%; p = 0.03). More patients had PDD <5 mm in cephalad adjacent discs compared to caudal adjacent discs (84.62% vs. 58.33%; p = 0.03). No significant difference was found comparing the proportion of caudal and cephalad adjacent discs that underwent degeneration (37.50% vs. 34.62%; p = 0.41). We further analysed the impact of different PDD cutoffs, varying from 2 mm to 6 mm, and found that a PDD of at least 4 mm significantly decreases the likelihood of adjacent disc degeneration (p = 0.03).

Conculsion

There was a significant increase in adjacent disc degeneration in patients undergoing ACDF when PDD was <5 mm. There was no difference in patient outcome between patients with two discs <5 mm away from the plate or patients with only one. A PDD of at least 4 mm decreased the likelihood of adjacent disc degeneration.

## Introduction

Anterior cervical discectomy and fusion (ACDF) is a common surgical method used to treat a variety of cervical spine diseases, including traumatic fracture dislocations. Although ACDF successfully returns stability and reduces symptoms related to the index disease, questions have been raised about the long-term effects of adjacent-level disc ossification and degeneration [1-3]. Because of the disturbance of spinal architecture and changed biomechanics, traumatic injuries to the cervical spine, such as fracture dislocations, pose special complications. Therefore, it is critical to comprehend the possibility of neighbouring-level degeneration and ossification after ACDF in the setting of severe damage to maximise patient outcomes and guide therapeutic decision-making.

Adjacent-level disc degeneration is a major clinical concern and frequently calls for further procedures, jeopardising long-term spinal health [4]. According to research on degenerative cervical disorders, one important factor influencing neighbouring-level degeneration following ACDF is the plate-to-disc distance (PDD). More specifically, in degenerative disorders, a PDD of less than 5 mm has been linked to an increased risk of neighbouring-level degeneration [5]. It is unclear, however, how these results relate to traumatic cervical spine injuries in general.

Different from degenerative disorders, the biomechanical effects of traumatic cervical spine injuries may change how the body reacts to surgical procedures like ACDF [6]. Traumatic injuries cause changes in the loading patterns and tissue responses by upsetting the normal spinal structure and biomechanics. As such, in contrast to degenerative disorders, the connection between surgical factors, such as PDD, and adjacent-level degeneration may be different in the setting of traumatic damage.

We reviewed the possibility of neighbouring-level disc degeneration and ossification after ACDF for traumatic neck fracture dislocations. The aim of this study is to assess the impact of PDD on adjacent-level disc degeneration in traumatic cervical spine injuries within two years of surgery.

## Materials and methods

The aim of this study was to assess the impact of PDD on future degeneration of the disc. We retrospectively reviewed imaging of patients’ cervical spines presenting with cervical fracture dislocations before and after surgery to measure PDD and disc degeneration.

We included patients who underwent ACDF surgery using an anterior plate for fracture dislocation of the cervical spine between 2017 and April 2023 at the Royal London Hospital, London, UK. ACDF for indications other than trauma and fusion without anterior plating were excluded. Two patients who had ACDF for cervical trauma also had underlying degenerative spinal conditions, and these patients were also excluded as the degenerative spinal conditions could prove to be a confounding factor.

**Figure 1:**
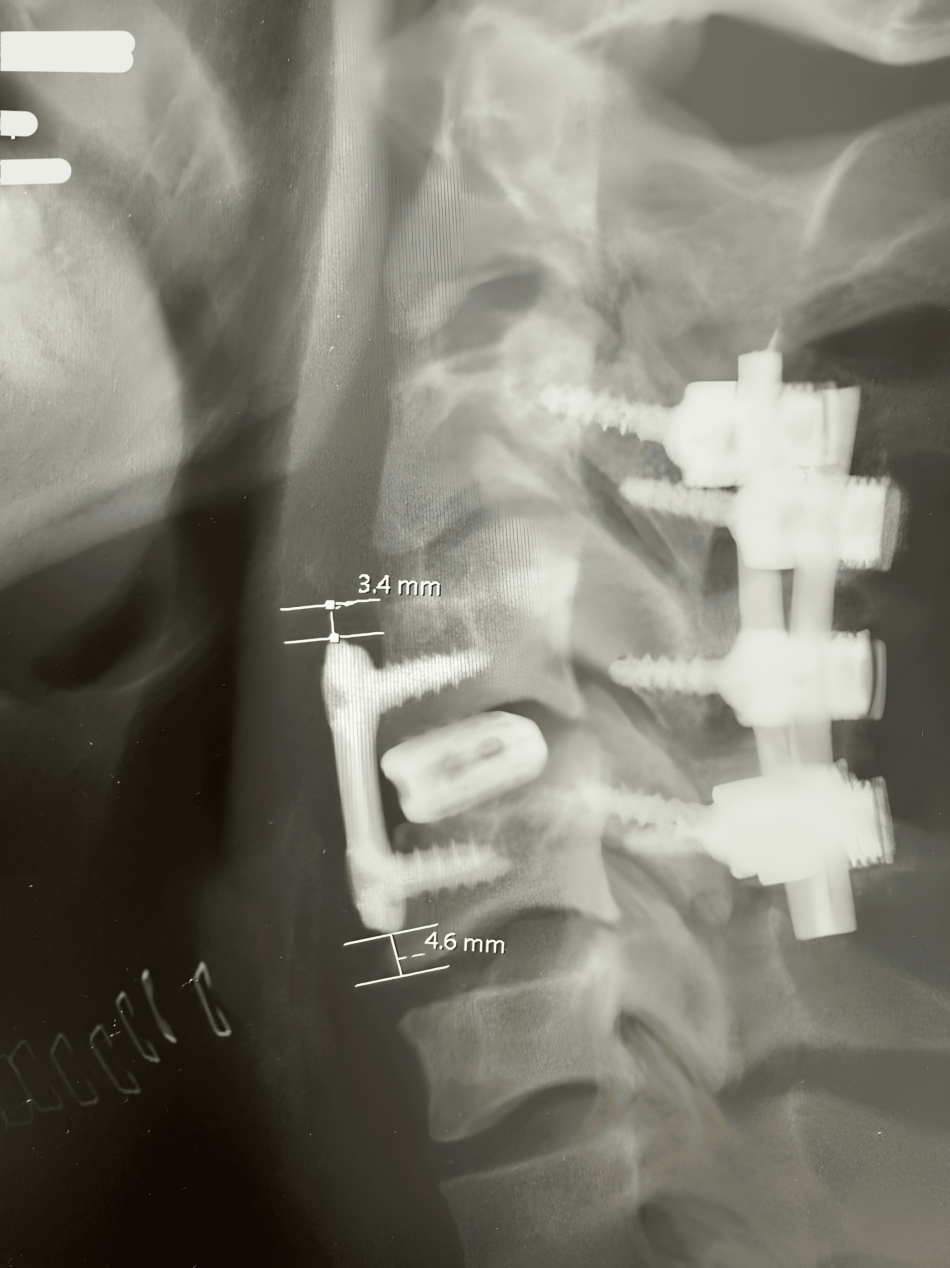
CT scan of the cervical spine after ACDF showing the method of calculating the PDD CT: computed tomography, ACDF: anterior cervical discectomy and fusion, PDD: plate-to-disc distance

We retrospectively reviewed images of the patients immediately after surgery and at least six months after surgery. CT scans and plain radiographs were used. The distance between the edges of the plate and the superior and inferior discs was measured using the scans obtained after surgery. To calculate the PDD, we drew a line of best fit at the vertebral body edge and at the closest end of the plate and then drew the line of shortest distance between the two (Figure 1).

The disc height was measured before surgery and at least six months after surgery. We used a similar method to calculate the disc height, drawing a line of best fit at the vertebral body edges and then drawing the line with the shortest distance between the two. The change in disc height was compared, and degeneration was assumed if there was a decrease in height greater than or equal to 30% and signs of a disc osteophyte complex [7,8]. The time between the scans that were used to compare degeneration of the discs varied between six months and two years (mean of 12.6 months, median of 12 months).

A total of 50 discs were included in the study. In particular, 84% of the discs included in the study were from male patients compared to 16% from female patients. The age range of the patients varied between 17 and 79. Twenty-four of the discs were cephalad, while 26 were caudal.

We first separated the discs into two groups. In Group A, the discs had a PDD greater than or equal to the cutoff. In Group B, the discs had a PDD smaller than the cutoff. Since the gold standard for ACDF is for the PDD to be equal to or greater than 5 mm, 5 mm was set as the cutoff [2,3].

We then evaluated different cutoff distances for a wider understanding and better optimisation of the impact of PDD on degeneration. These distances ranged between 2 mm and 6 mm. The percentage of discs that underwent degeneration in these groups was also compared. All of our measurements were performed by two separate authors, and the results were compared. While there was some small variation in the measurements, both authors agreed with the categories the measurements resided in.

Our data are presented as counts and percentages (n (%)). In both parts of the study, a Z-test was used to evaluate whether there was any statistically significant difference in disc degeneration when the PDDs were above or below the cutoff distances. The Z-test was chosen as it allows for comparison of the difference between two proportions and is suitable for use in small sample sizes. We set a p-value of 0.05 as the boundary. MS Excel (Microsoft Corp., USA) was used to calculate this.

## Results

Results for the 5 mm cutoff

The results comparing Groups A and B are shown in Table 1. There is a statistically significant increase in adjacent-level disc degeneration when the PDD is less than 5 mm compared to when the PDD is equal to or greater than 5 mm (p = 0.03593, z = -1.8001).

**Table 1:** Number of discs with and without degeneration and % of discs with degeneration in Groups A and B

	No. of discs with degeneration	No. of discs without degeneration	Total no. of discs	% of discs with degeneration
Group A	2	11	13	15.38
Group B	16	21	37	43.24

The proportion of caudal and cephalad discs that had a PDD of less than 5 mm and the percentage of discs with degeneration are shown in Table 2. While there was a statistically significantly larger proportion of cephalad discs with a PDD <5 mm (p = 0.03846, z = -2.0679), there was no significant difference when comparing the percentage of discs with degeneration.

**Table 2:** Comparison of the percentage of caudal and cephalad discs with a plate-to-disc distance (PDD) <5 mm and the percentage of discs with degeneration.

	% of discs with a plate-to-disc distance <5 mm (%)	% of discs with degeneration (%)
Caudal discs	58.33	37.50
Cephalad	84.62	34.62

Results for cutoffs of 2-6 mm

**Figure 2:**
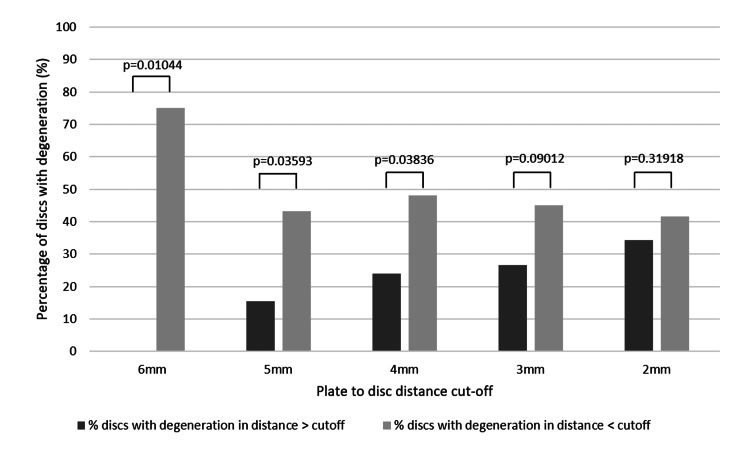
Comparison of the % of discs with degeneration in Groups A and B at different cutoffs.

The results evaluating and comparing the impact of different cutoffs on the percentage of adjacent-level disc degeneration are shown in Table 3 and Figure 2. Starting at the 4 mm, there is a statistically significant difference in disc degeneration when the PDD is below the cutoff (p = 0.03836, z = -1.7678).

**Table 3:** Comparison of the number of discs with and without degeneration and % of discs with degeneration in Groups A and B at different cutoffs.

Cutoff distance (mm)		No. of discs with degeneration	No. of discs without degeneration	Total no. of discs	% percentage of discs that underwent degeneration (%)
6	PDD ≥ 6 mm	0	8	8	0.00
	PDD < 6 mm	18	24	42	75.00
5	PDD ≥ 5 mm	2	11	13	15.38
	PDD < 5 mm	16	21	37	43.24
4	PDD ≥ 4 mm	6	19	25	24.00
	PDD < 4 mm	12	13	25	48.00
3	PDD ≥ 3 mm	8	22	30	26.67
	PDD < 3 mm	9	11	20	45.00
2	PDD ≥ 2 mm	13	25	38	34.21
	PDD < 2 mm	5	7	12	41.67

**Figure 3:**
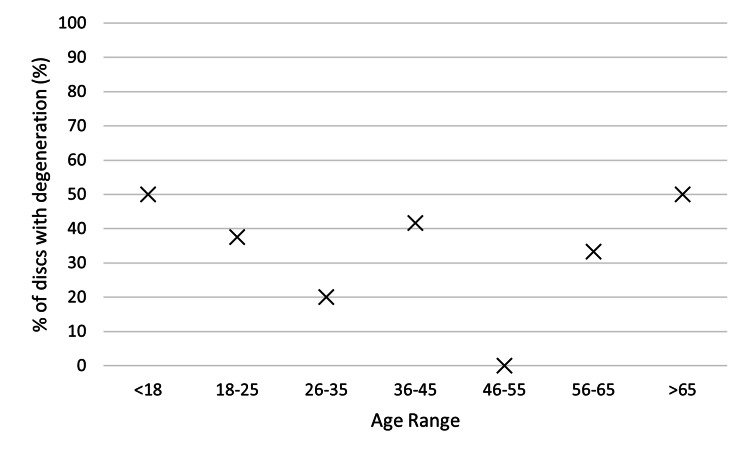
Graph comparing the age range of the patients and % of discs with degeneration.

**Figure 4:**
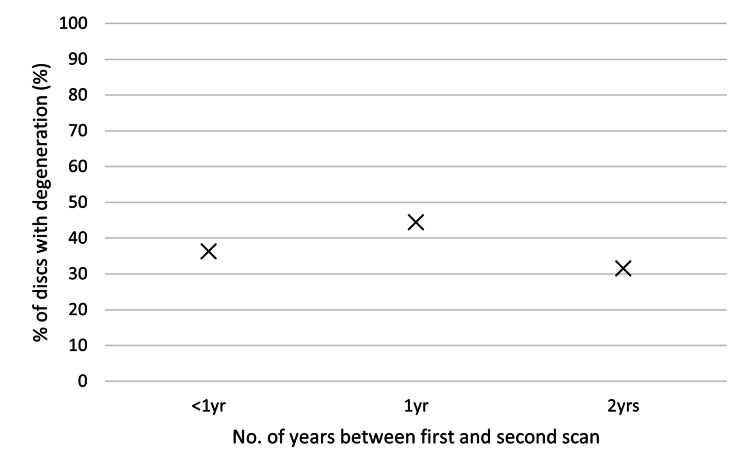
Graph comparing the % of discs with degeneration and the number of years between the first and second scans.

**Figure 5:**
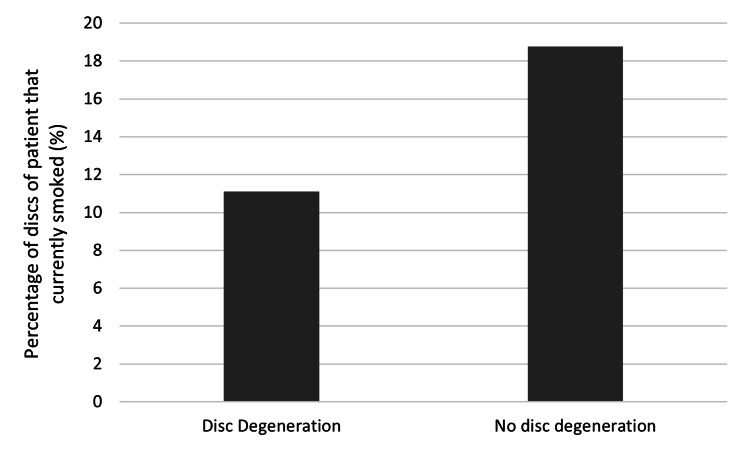
Graph comparing the percentage of discs of patients that currently smoked that degenerated vs. did not degenerate. P-value = 0.23885, no significant difference between the two groups.

**Figure 6:**
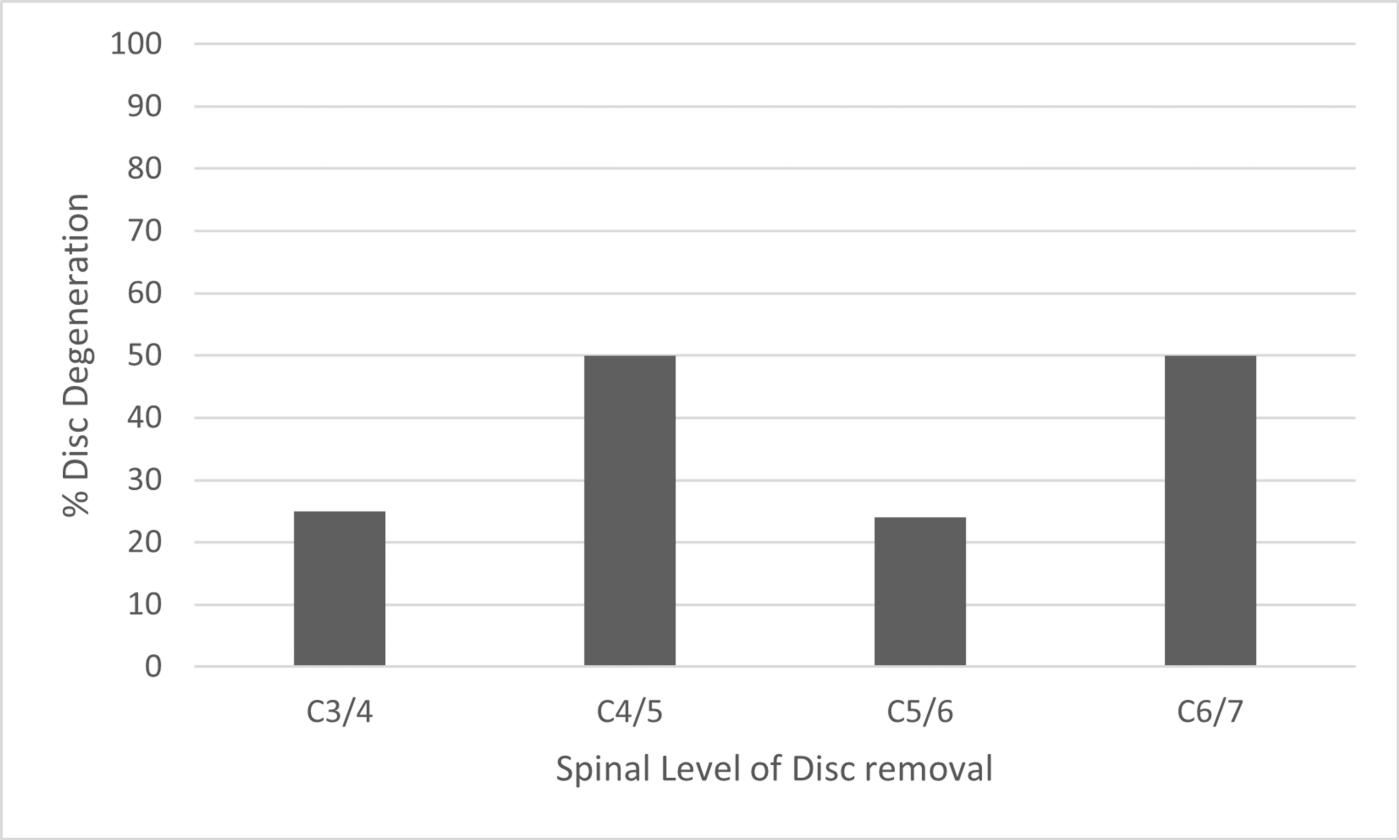
Graph comparing the spinal level of the disc removal with the % of adjacent level discs that degenerated.

Gender, age, smoking status, and the number of years between the first and second scans did not have any statistically significant impact on disc degeneration (see Table 4, Figure 3, Figure 4, and Figure 5). The level of the discs was also noted; however, once again, there was no statistically significant impact on disc degeneration (Figure 6).

**Table 4:** Comparison of gender to % of discs with the plate-to-disc distance (PDD) less than the cutoff and % of discs with degeneration.

	Male	Female	p-value
% of discs that degenerated	33.33%	50.00%	0.36812
% of discs that had a disc-to-plate distance <6 mm	83.33%	75.00%	0.57548
% of discs that had a disc-to-plate distance <5 mm	71.43%	75.00%	0.83366
% of discs that had a disc-to-plate distance <4 mm	51.16%	50.00%	0.90448
% of discs that had a disc-to-plate distance <3 mm	42.86%	37.50%	0.77948
% of discs that had a disc-to-plate distance <2 mm	26.19%	16.67%	0.40654

## Discussion

Previous research demonstrated the increased risk of adjacent discoosteophyte complexes and degeneration after ACDF in patients with degenerative disease when PDD was less than 5 mm [2,9]. The results of this study show that there is also an increased risk of adjacent-level ossification after ACDF in trauma patients. Further investigation into the impact of PDD on disc degeneration in our study also showed that a distance of at least 4 mm could significantly decrease the risk of adjacent-level disc degeneration. To our knowledge, this is the first study to evaluate this threshold specifically in a trauma context without the confounding effects of pre-existing degenerative disease.

Former studies often focused on the use of ACDF on degenerative spinal conditions and, as a result, were unsure whether the degenerative changes in the superior and inferior discs were a result of the surgery or of their patients’ underlying condition [2,9-11]. By excluding any patient with degenerative disease, this study was able to eliminate this confounding factor and therefore showed the direct impact of plate placement on disc degeneration.

We also found that caudal discs were more likely to undergo degeneration than cephalad discs. While Park et al. [2] previously observed this pattern, they attributed it to a higher proportion of caudal plates being positioned less than 5 mm from the adjacent disc. In our cohort, plate position was more evenly distributed, suggesting that other factors may contribute to this trend. However, this remains a hypothesis, and larger studies are required to explore this observation further.

Although we observed a strong correlation between reduced PDD and increased degeneration, the mechanism behind this remains uncertain. Finite element studies have not consistently demonstrated significant differences in disc stress based on PDDs [12], while other authors have suggested that close plate positioning may cause inflammation or mechanical irritation of the anterior disc space [2]. Our data do not directly assess the causes behind disc degeneration, and further research is needed to clarify the causal mechanisms. Clarifying these mechanisms can help with the creation of customised rehabilitation plans meant to protect the spinal health of these susceptible patients.

This study has some limitations. Most notably, the sample size of 50 patients limits statistical power and generalisability. While the findings are clinically relevant, they should be interpreted cautiously. This study is also limited by its retrospective design, which may introduce selection bias and limit control over follow-up duration, imaging assessments, and patient comorbidities. While all patient data were systematically collected and radiological measurements were independently verified to minimise potential confounding factors, we were unable to take into account the impact of all of the patient's comorbidities. With regard to the follow-up, we can note that the range in the follow-up durations of the patients was large. While we have tried to mitigate this by comparing the impact of follow-up duration against disc degeneration (Figure 4), this still remains a confounding factor. Furthermore, this study relies primarily on radiological outcomes, as clinical follow-up data were limited due to non-response from many patients, which may restrict the interpretation of functional relevance. Additional prospective studies with larger cohorts and longer and more standardised follow-up are needed to confirm the optimal PDD threshold and to explore the biological and mechanical contributors to adjacent segment degeneration.

## Conclusions

Our study shows that there may be a statistically significant increase in adjacent disc degeneration when the plate is positioned at a distance less than 5 mm from the intervertebral disc. Furthermore, on further analysis, it suggests that maintaining a distance of at least 4mm between the plate and the adjacent disc could significantly reduce the likelihood of disc degeneration. Despite the limitations, our findings may have practical implications. Ensuring adequate plate positioning during surgery could play an important role in improving long-term patient outcomes and reducing the burden of postoperative complications. Further research should continue to investigate the effects of PDD on disc degeneration and focus on investigating the clinical outcomes of patients. Investigation into the mechanism behind the disc degeneration may also help us better understand the causes. 
